# Children’s perceptions about medicines: individual differences and taste

**DOI:** 10.1186/s12887-015-0447-z

**Published:** 2015-09-21

**Authors:** Julie A. Mennella, Kristi M. Roberts, Phoebe S. Mathew, Danielle R. Reed

**Affiliations:** Monell Chemical Senses Center, 3500 Market Street, Philadelphia, PA 19104-3308 USA

**Keywords:** Children, Compliance, Genetics, Medication, Taste

## Abstract

**Background:**

Bitter taste receptors are genetically diverse, so children likely vary in sensitivity to the “bad” taste of some pediatric formulations. Based on prior results that variation in a bitter taste receptor gene, *TAS2R38*, was related to solid (pill) formulation usage, we investigated whether this variation related to liquid formulation usage and young children’s reports of past experiences with medicines and whether maternal reports of these past experiences were concordant with those of their children.

**Methods:**

We conducted retrospective interviews of 172 children 3 to 10 years old and their mothers (*N* = 130) separately in a clinical research setting about issues related to medication usage. Children were genotyped for the *TASR38* variant A49P (alanine to proline at position 49). Children’s responses were compared with their *TAS2R38* genotype and with maternal reports.

**Results:**

Children (>4 years) reported rejecting medication primarily because of taste complaints, and those with at least one sensitive *TAS2R38* allele (AP or PP genotype) were more likely to report rejecting liquid medications than were those without a taster allele (AA genotype; χ^2^ = 5.72, df = 1, *p* = 0.02). Children’s and mothers’ reports of the children’s past problems with medication were in concordance (*p* = 0.03).

**Conclusions:**

Individual differences in taste responses to medications highlight the need to consider children’s genetic variation and their own perceptions when developing formulations acceptable to the pediatric palate. Pediatric trials could systematically collect valid information directly from children and from their caregivers regarding palatability (rejection) issues, providing data to develop well-accepted pediatric formulations that effectively treat illnesses for all children.

**Trial Registration:**

Clinicaltrials.gov protocol registration system (NCT01407939). Registered 19 July 2011.

## Background

Most children, at some point in their lives, are given medicine to treat an illness or disease, and some will reject it. A variety of factors, including the child’s age, body size, mechanics of swallowing [1], and taste preferences [2] affect acceptance of medicine. While factors inherent to the child cannot be changed, the formulation of the medicine can be. Pediatric medications come in several oral formulations (liquid, tablet or pill) and contain flavors and excipients (e.g., sweeteners), which can cater to the pediatric palate [2]. However, while solid oral dosage forms (pills) have the advantage of encapsulating the taste of active pharmaceutical ingredients (so pills are less bitter and less irritating than liquids), some children have difficulty swallowing them, and fixed doses are often impractical for body-weight-based dosages. Moreover, many drugs have not been clinically tested in infants and children and thus lack appropriate pediatric formulations [3, 4], leading many to recognize the general need for better medicines for children worldwide.

Children cannot benefit from medicines they will not take [5]. “Taste” is often cited as a primary issue for noncompliance [5], based on a variety of questionnaire-based survey and phone interview studies of parents [6–8], physicians [9, 10], and health care personnel [9], but studies rarely asked children directly about their likes and dislikes of medications (but see ref. [11]), and few have determined whether mothers’ reports about their children’s acceptance or rejection of the medicine match those of their children. In the present study, we used a clinical research setting to separately interview directly both children and their mothers. We probed whether children can respond to open-ended questions about past experiences with medicines to determine whether their reports are concordant with those of their mothers. We also genotyped the children for a known bitter taste receptor gene but acknowledge that bitter taste is not the sole culprit of the type of bad taste of medicines, since many drugs can irritate the throat or mouth and contain unpleasant volatiles [2].

Because not all children reject medicines, genetic variation in taste receptor biology may explain some of these individual differences. During the past decade, research has reported 25 members in the TAS2R family of bitter-taste receptors [12]. These receptors are selectively sensitive to particular compounds and are genetically extremely diverse [12]. The most studied bitter taste receptor gene, *TAS2R38,* has several forms [13, 14]. People who are homozygous for the insensitive form (AA) typically cannot taste the bitterness of its ligands, including a medication to treat hyperthyroidism, propylthiouracil (PTU or PROP) [13, 14]. The phenotype–genotype relationship for this receptor varies with age such that children with the bitter-sensitive genotypes (AP, PP) are more sensitive to the bitter taste of this medicine than are their parents with the same genotypes [15, 16]. Further, recent evidence suggests that variation in bitter taste receptor genotype may be related to medication acceptance among children. That is, a retrospective analysis found that children with bitter-sensitive (homozygous PP and heterozygous AP) *TAS2R38* genotypes were more likely to have taken medication in a solid formulation than were children with the bitter-insensitive (AA) genotype [17], perhaps because their bitter sensitivity makes them more motivated to take pills or tablets.

In this study, we queried a large and diverse group of children (*N* = 172) and their mothers about past experiences with medicine focusing on liquid formulations since this is the most frequently experienced formulation type taken by children of this young age group. Children were genotyped for the *TAS2R38* A49P allele to test the hypothesis that variation in this bitter taste receptor gene may explain individual differences in some “taste” issues encountered in using liquid formulations and their reports of past experiences with medicines.

## Methods

### Participants

Participants were healthy 3- to 10-year-old children and their mothers who participated in two research studies on bitter taste perception [15, 18]. During the telephone interview, the mothers were given detailed descriptions of the procedures for the present study but were not told the goals of the study or hypotheses being tested. Women who were diabetic, pregnant, or lactating were not eligible, and pregnancy tests were conducted on the day of testing to confirm they were not pregnant. Children who were on any medications that may alter taste sensitivity were excluded from the studies. All children were reported to be healthy by their mothers.

### Ethics committee approval

All procedures were approved by the Office of Regulatory Affairs at the University of Pennsylvania, Protocol Number 809789. Written informed consent was obtained from a parent of each child, and assent was obtained from each child 7 years of age and older. The study was registered on ClinicalTrials.gov Protocol Registration System (NCT01407939).

### Procedures

Mothers and children were queried separately in private testing rooms. Mothers completed questionnaires regarding demographics and race (assigned per US Census categories) and were asked individually about their child’s overall medication history, including types of formulations (e.g., liquids, drops, pills or tablets, nasal sprays), flavor preferences, and past problems. Children were also asked directly and privately (in a separate testing room without the presence of the mother) about their past experiences of taking medicines: whether they were ever given medicine to drink, chew, or swallow; if so, whether there were any medicines they would not take; and if so, why they refused.

### Genotyping methods

A saliva sample was collected and genomic DNA was extracted from it following the directions of the manufacturer (Oragene, DNA Genotek, and Canada). The *TAS2R38* A49P alleles (rs713598; accession no. AF494231) were genotyped using dye-based primers and probes (Life Technologies, Grand Island, New York). Children were identified as bitter-insensitive homozygous (AA), bitter-sensitive homozygous (PP), or heterozygous (AP) [13]. Although there are three common variant sites in this gene, we chose to group children by the first one (A49P, rs713598) because it explains most of the individual differences in the taste response [16, 19] and is a proxy for other variants due to linkage disequilibrium [20]. Genotyping quality steps included assaying known control samples, assaying 10 % of samples in duplicate, and establishing that genotypes were in Hardy-Weinberg equilibrium.

### Statistical analyses

All analyses were conducted using Statistica (version 12; StatSoft, USA). ANOVAs determined whether children grouped by formulation acceptance varied by age. Nonparametric analyses assessed whether there were associations 1) between *TAS2R38* genotype and reported problems with liquid medications and 2) between responses of children and their mothers. Genetic analyses were conducted assuming a dominant model [13, 16, 18] in which children with one or two bitter sensitive alleles were grouped and compared to children who were homozygous for the insensitive allele. Summary statistics are means ± SEM or percentage of group.

## Results

The mothers averaged 33.9 ± 0.7 years old (*N* = 130), and the children (*N* = 172) were between the ages of 3 and 10 years. Included in the sample were 94 singletons, 31 sibling dyads, 4 sibling triads, and 1 sibling tetrad. As shown in Table 1, children’s race/ethnicity, family yearly income, and mothers' highest education level, based on maternal reports, reflected the racial and socioeconomic diversity of the Philadelphia area [21]. Duplicate genotyping assay results matched in every case and genotypes were in Hardy-Weinberg equilibrium [χ2_(2)=_2.45, *p* = 0.29]. Genotypes of three children could not be obtained even after multiple attempts.

**Table 1:** Subject demographics

Measure	Data
Children (*N = 172*):	
Sex (girls, boys)	97 girls, 75 boys
Age, years [mean ± SEM (n)]	7.8 ± 0.1 (172)
Race/ethnicity [% (n)]	
White	12.8 % (22/172)
Black	56.4 % (97/172)
Hispanic	14.5 % (25/172)
Asian	1.2 % (2/172)
Other/more than one race	15.1 % (26/172)
*TAS2R38*, A49P genotype [ % (n)]^a^	
AA	29.6 % (50/169)
AP	45.6 % (77/169)
PP	24.8 % (42/169)
No. children who did not answer questions regarding medication usage [% (n)]	11 % (19/172)
Non-Responders’ Genotype	
AA	15.8 % (3/19)
AP/ PP	84.2 % (16/19)
Mothers (*N = 130*)^b^ *:*	
Age, years [mean ± SEM (n)]	33.9 ± 0.7 (130)
Family Yearly Income, [% (n)]	
< $35,000	62.3 % (81/130)
$35,000–$75,000	26.2 % (34/130)
> $75,000	11.5 % (15/130)
Highest Education Level, college graduate, [% (n)]	48.5 % (63/130)

^a^Data from 3 children were refractory to genotyping

^b^Mothers of 94 singletons, 31 sibling pairs, 4 sibling triads, and 1 sibling tetrad

Mothers reported that the children had last been given medication within the past 6.1 ± 0.5 months (range: <1-36 months): cold and pain remedies (98.3 %), antibiotics (52.9 %), antihistamines (26.2 %), anti-asthmatics (11.0 %), gastrointestinal (4.7 %), antifungal (4.1 %), and psychiatric (2.9 %). All had prior experience with liquid formulations. Cherry, bubble gum, and grape were reported to be the children’s favorite flavorings. Regardless of their child’s age, most mothers preferred pediatric liquid formulations (63.4 %), followed by chewable tablet (19.8 %) or gummy (9.3 %) formulation (Table 2).

**Table 2:** Children’s medication history as reported by Mothers^a^

Questions	% (n/N)
Child has taken medications	100 % (172/172)
Liquid drops or liquids	100 % (172/172)
Chewable	34.3 % (59/172)
Nasal sprays	18.0 % (31/172)
Pills or tablets	19.8 % (34/172)
Child had problems taking medication	48.3 % (83/172)
Liquid drops or liquids	41.9 % (72/172)
Chewable	16.1 % (9/56)^b^
Nasal sprays	45.2 % (14/31)
Pills or tablets	32.4 % (11/34)
Preferred pediatric formulation	
Liquid	63.4 % (109/172)
Chewable tablet	19.8 % (34/172)
Gummy	9.3 % (16/172)
Pill/tablet	2.3 % (4/172)
Strips	1.1 % (2/172)
No preference/other	4.1 % (7/172)

^a^If mother had multiple children in the study, she reported which formulation she most preferred for her children

^b^Data missing for three mothers for this entry

Not all children answered the questions. Of the 172 children, 19 (11.0 %) did not respond when asked if they had ever refused medication (Table 1). These 19 children were significantly younger (6.2 ± 0.4 years) than the 153 children who did respond to the questions (8.0 ± 0.1 years; F(1, 170) = 18.69; *p* < 0.0001). The vast majority of the non-responders had bitter sensitive genotypes (84.2 % were AP/PP; 15.8 % were AA). None of the children who were younger than 4 years of age responded to the questions.

Of the 153 children who responded to the questions, 89 (58.2 %) reported refusing to take medications, and 86 (96.6 % of those who reported refusal) responded when asked why they had refused. We found mother-child concordance (*N* = 153 dyads) in reports of past problems taking medications (χ^2^ = 4.96, df = 1, *p* = 0.03). About half of the mothers (48.3 %; Table 2) and children (58.2 %; Table 3) reported such problems. The primary reason children gave for rejecting medicine was “taste” complaints (Table 3).

**Table 3:** Reasons given by children for refusing medications

Taste/flavor, 84.9 % (73/86)	
“Nasty”/“Nasty taste” (*n* = 32)	“Doesn’t taste good”
“Yucky” (*n* = 4)	“Taste like fish”
“Bitter” (*n* = 3)	“Don’t like grape”
“Tastes horrible” (*n* = 2)	“Sour/salty taste”
“Gross/tastes gross” (*n* = 2)	“Bitter cherry/ear wax taste”
“Tastes ugh” (*n* = 2)	“Doesn’t taste like cranberries”
“Bad taste” (*n* = 2)	“Only like blueberries”
“Icky taste later”	“Fruit flavor, only bubble gum flavors”
“Nasty after taste”	“Tastes nasty, only like bubble gum”
“Tastes old”	“Nasty, doesn’t like cherry”
“Tastes like poison”	“Tastes nasty/doesn’t like color or flavor”
“Don’t like taste”	“Too hard”
“They have vegetables inside and don’t taste good”	“Mom puts it in salty water”
“Tastes like alcohol”	“Tastes like salt water”
“Tastes like diet”	“Tasted horrible and scared to swallow”
“Hated taste”	“Tastes too sour, old people like them”
“Disgusting”	
Problems with swallowing or choking, 8.1 % (7/86)	
“Hard to swallow” (*n* = 2)	“Scared to choke”
“Couldn’t swallow and choked on it”	“Have to drink water to swallow them”
“Gag, can’t chew, hard to swallow”	“Afraid because little boy on TV choked from pills”
Consequences of taking medicine, 2.3 % (2/86)	
“Allergic”	“Makes me have headaches”
Combination/other, 4.7 % (4/86)	
“I don’t know” (*n* = 2)	“I don’t know what to do with them”
“Medicine is for grownups”	

Responses are *n* = 1, except as noted

Reports of medication compliance were related to bitter receptor genotype. More children with at least one sensitive *TAS2R38* allele (AP, *N* = 77; PP, *N* = 42) reported having problems accepting liquid formulations (48 % with AP/PP, *N* = 57/119) than did those with no bitter alleles (28 % with AA, *N* = 14/50; χ^2^ = 5.72, df = 1, *p* = 0.02). Of those children who had been offered pills (*N* = 34), there was no difference in age between those who rejected (8.8 ± 0.4 years, *N* = 11) or accepted (8.7 ± 0.3 years, *N* = 23) them (F(1,32) = 0.027; *p* = 0.87). More than half of these children were trained to take pills (58.8 %; *N* = 20/34), as reported by their mothers. One-third of these children (35 %; *N* = 7/20) had problems swallowing or rejected the pills despite training. While this small sample size precludes statistical conclusions, we found that 75 % (15/20) of children with at least one bitter-sensitive allele (AP/PP) reported having taken a solid formulation compared to 57 % (8/14) of children with no bitter-sensitive alleles (AA).

## Discussion

Based on prior results that variation in a bitter taste receptor gene, *TAS2R38*, was related to solid (pill) formulation usage [17], we interviewed children and their mothers separately and included questions about liquid formulation usage and memories of past experiences with medicines, and then determined if children’s responses were related to their *TAS2R38* genotype and with responses of their mothers. Mothers reported having problems administering all types of oral formulations to their children, and they and their children reported rejecting medications primarily for “taste” reasons. However, not all children (especially those <4 years old) responded to open-ended questions regarding past use of medication, highlighting limitations in collecting such information in this manner from younger children. Consistent with prior reports [10, 22–24], liquid formulations were preferred by mothers but were most reported by children as being problematic to take. Such findings reflect children’s biology: research on children of the age range in the present study (3–10 years) has repeatedly revealed that they reject bitter tastes [13, 15, 16] and avoid unpleasant flavors and textures [2] but favor sweet (pleasant) tastes [18, 25, 26]. The child’s most preferred levels of sweetness and sensitivity to bitterness do not go through pronounced changes until mid-adolescence, achieving levels measured in adults [16, 26].

Some mothers attempted to train their children to swallow pills, with only moderate success. Some children voiced concerns about taking pills and fear of choking [27]. Children who had successfully taken a solid dosage form averaged 9 years of age, a finding remarkably consistent with data derived from pharmacy dispensing records in the Netherlands [4]. Like teenagers and adults, older children vary greatly in biomechanics of swallowing and ability to swallow tablets and capsules [1], despite behavioral training [27, 28]. Therefore, offering medicines in pill form to children is only partially successful even for older children.

Not only are some children more sensitive to bitter tastes than are adults despite similar genetics [15, 16], but we found that some children were genetically more sensitive than others, and such differences are related to liquid medication usage and acceptance. In the present study, children who had at least one sensitive allele (PP or AP) of the bitter taste receptor gene *TAS2R38* were more likely to report rejecting liquid medications than were children with the insensitive (AA) allele, extending our previous findings that this taste genotype is associated with experience of solid medicine formulations [17]. Unlike this prior study where many of the 3- to 10-year-old children (*N* = 138) had taken medicine in solid form [17], few children (*N* = 34) in the present study had done so. Nevertheless, we have observed the relationship between *TAS2R38* genotype and issues related to medications in two independent populations of children ([17]; present study). Although the *TAS2R38* receptor gene is unlikely to be sensitive to all bitter compounds found in medications, its alleles may be a proxy for general taste ability [29], are related to acceptance of other bitters such as those found in vegetables [30] and are associated with individual differences in other aspects of taste biology (e.g., sweet preference) [13]. Also, because bitter taste receptor genes occur in linked clusters [31], genetic variation in this receptor may be related to variation in other receptors. Future work should also relate variation in medication acceptance among children to polymorphism of other taste receptor genes, including those related to sweet taste.

The present study focused on children’s and mothers’ reports of past experiences with medications, rather than assessing taste rejection or actual compliance with a specific medication. While not all children can provide information on the sensory acceptance of medications, we found concordance between reports by children and their mothers regarding medication usage, indicating both the ability of children to report on their own experiences and the reliability of their mother’s reports for children who are not able to respond themselves. Children’s perceptions, as well as those of their caregivers, are valuable and, as illustrated herein, highlight the formidable task faced by health professionals and parents to provide oral dosage formulations that children like or accept its taste. An estimated 40 % of the world’s children are at increased risk for avoidable adverse events such as suboptimal dosing and lack of adherence to medication regimens [3, 10, 32]. How much suboptimal dosing arises solely from the bad taste of medicine is unknown, but these data suggest that prospective studies are needed to understand the role of individual differences in taste acceptance of individual medicines. Our data point toward the feasibility of gathering such information from children and, for very young children, from their mothers regarding experiences with medications. However, it is important to note that for foods, mothers are more accurate in the types of foods that are disliked by their children than those that are liked [33]. Thus, it may be if one is interested in children's dislikes of particular medicines, maternal reports might be suitable but if one is interested in their likes, applying age-appropriate sensory methodologies rather than maternal reports may be more appropriate [2].

Future pediatric clinical trials thus could systematically collect data regarding taste acceptance/palatability of particular medicines directly from children and their caregivers (see [11]). Such data, combined with information on the type of formulation, types of excipients, and methods of administration [34], will help develop and validate nonproprietary methods to assess behaviors associated with concepts such as “acceptance”, “rejection” and “palatability”. These methods need to be sensitive to the cognitive limitations of children of varying ages (see ref. [2] for discussion). The ultimate goal is to develop well-accepted pediatric formulations that effectively treat illnesses for all children.

## Conclusions

In this study, children reported rejecting medication primarily because of taste complaints, and those with at least one sensitive *TAS2R38* allele (AP or PP genotype) were more likely to report rejecting liquid medications than those without a taster allele. Thus, individual differences in taste responses to medications highlight the need to consider children’s genetic variation and their own perceptions when developing formulations acceptable to the pediatric palate. Mothers’ and children’s reports of children’s past problems with medication matched, indicating that pediatric trials could systematically collect information directly from either children or their caregivers regarding issues related to acceptance or rejection of medicines, providing data to develop well-accepted pediatric formulations that effectively treat illnesses for all children.
